# On the Coloring Matter of the Black Bronchial Glands, and of the Black Spots of the Lungs

**Published:** 1814-02

**Authors:** George Pearson


*For the Medical and Physical Journal.*

### *On the coloring Matter of the black Bronchial Glands, and of the black Spots of the Lungs; by* George Pearson, M.D. F.R.S.

IN the adult human animal, the glands designated *bronchial* are generally of a black or dark-blue color. These organs, which are allowed to be lymphatic glands, as is well known, are situated at the root of the lungs externally, within cellular membrane, near the bifurcated trachea; as well as internally on or near the large branches of the bronchi.

At the age of about twenty years, the lungs have a mottled or marbled appearance, from black and dark-blue spots, lines, and points disseminated immediately under the transparent pulmonary pleura. As hath been repeatedly observed, the lungs generally become more dark-colored proportionately to their age. Accordingly, at upwards of sixty-five five or seventy years of life, they often appear almost uniformly black, from the number and congeries, or coalescence of the maculæ, points, and lines, just mentioned. Throughout the whole interior substance of the lungs, the black spots are seen in a great measure corresponding to the external appearance.

I do not find that any observations and experiments have been made to determine the nature or cause of the black color above described of the pulmonary organs. It is true, a conjecture has been proposed, that sooty matter taken in with the air may be the occasion of the color of the lungs; and that the color of the glands is occasioned by a peculiar secretion. But the former conjecture has been supposed to be satisfactorily refuted by the absence of the appearance in question among brute animals; as also by its presence in persons who breathe the air of the provinces at a great distance from towns, or places of great consumption of coal; and the latter conjecture is palpably erroneous, because the bronchial glands, of which I am speaking, are not organs of secretion, but of conveyance of lymph.

The course of investigation, in which I have long been engaged, to improve the pathology of pulmonary consumption, led me to some experiments and observations on the subject now stated, which I respectfully submit to the consideration of the Society.

After cutting away the cellular membrane surrounding the black glands, and washing them till the water was no longer colored, I subjected them to examination.

1. On pressure between the fingers, to burst the investing coat, a black fluid issued which stained the skin, which rendered water black, and which did not alter in color, or apparently dissolve, even at a boiling temperature, either in water or in concentrated muriatic or nitric acids.

2. On breaking down the structure, and triturating in a glass mortar a number of these glands with a small proportion of water, a thick black liquid was produced, which was decanted from off many membranous and fibrous masses. But after repeated affusions and trituration, I could not deprive these masses of their black color, although the water was at last scarcely tinged: it was only by dissolution in caustic potash lye, or in nitric and muriatic acids, that I could totally separate the black matter from the animal substance to which it seemingly adhered. After repose, a black sediment took place in the waters of elutriation, as well as from the alkaline, and the acid dissolution, which on decantation and evaporation to dryness, afforded the deposit in the state of a black powder.

The texture and proportion of the tinging matter of the glands was very different in different subjects, whether the lungs, to which they belonged, were in a healthy or diseased condition. In persons of about eighteen to twenty years of age, some of the bronchial glands contained no tinging black matter at all, but were of a reddish color; others were streaked, or partially black; and others were quite black, or of a dark-blue color.

3. By boiling the black glands in lye of caustic potash, their structure was destroyed, and a turbid black liquid was produced, from which, on standing during several days, a copious black sediment took place; but the liquid still remained black, after remaining at rest a month, much of the tinging matter continuing suspended. By dilution with water, this matter deposited in a clear liquid.

4. By liquid muriatic acid, of the specific gravity 1.170, the bronchial glands were dissolved at a boiling temperature, affording a turbid black liquor; but on repose, an abundant black deposit took place from a clear yellow liquid, as well as a quantity of the same matter appearing on its surface. On separating this precipitate, and evaporating to dryness, it became a black powder.

5. Nitric acid liquid, of the specific gravity 1.500, most speedily dissolved the substances under examination, affording a clear yellow liquid with abundance of black matter on its surface. The separation of this coal-like matter is effected most easily with this acid.

It may not be quite superfluous to mention, that from the bronchial glands, which were not at all colored with black matter, but were merely red, no black matter was separable by the above-mentioned acid and alkaline solvents.

I next examined the black and blue coloring substance of the surface and interior parts of the lungs.

1. A portion of the lungs beautifully marked with a crowd of these colored areolæ, spots, points, and lines, after being washed till it no longer imparted a bloody tinge to water, still retained these appearances undiminished. On pressure of these colored parts between the fingers, the skin was sometimes stained of a black color.

2. On treating the substance of the lungs, abounding in the above-described black and blue parts, in the same manner as the bronchial glands just related, with water, with liquid caustic potash, with muriatic and with nitric acids, similar phenomena, with specially the separation of black matter, was observed. The proportion, however, of this black substance was very much smaller to the animal matter dissolved, than in the former cases.

3. To

3. To show that the black matter is of the same kind in the differently figured black parts of the lungs, it may be worth while to relate a particular experiment.

A thin slice of about three inches square of the surface of lungs exhibiting black lines, maculæ, and points under the transparent pleura, was put into a vessel containing three ounces measure of liquid nitric acid. On this it floated, but all of it was presently dissolved, except the colored parts, which retained their respective forms till the vessel was shaken: then they were destroyed, and afforded a coating or stratum of black matter on the surface.

Having collected an adequate quantity of the coloring matter in a powdery state, for examination, I performed a number of experiments, but it seems needless to relate more than a few decisive ones.

1. A little of the well exsiccated black powder being sprinkled upon fused nitrate of potash, deflagration took place as with charcoal of wood, or with soot.

2. The same phenomenon occurred with melted oxymuriate, or chlorite of potash, but at a much lower temperature,

3. The deflagration with nitrate of potash, and also with chlorite of potash, was produced, in a suitable glass vessel, to collect the compounded gas; which was received into lime water, and found to be charcoal acid.

4. A little of the black powder was very easily ignited upon a plate of platina, and was speedily burnt off, smelling like burning animal matter, and leaving a minute residue, sometimes of reddish powder and at other times of white.

5. The powder was ignited in a green glass tube closed at one end, and kept red hot from ten to fifteen minutes, the open extremity being slightly stopt with a clay lute to prevent the admission of air. A white vapor with water was discharged, and a minute portion of animal empyreumatic oil was condensed in the upper part of the tube. There remained on cooling, a fine black powder, which in different trials lost $\frac{1}{7}$ to $\frac{1}{4}$; i. e. about $\frac{14}{100}$ to $\frac{25}{100}$ of the original weight of the substance.

6. On treating the coaly powder by exposing it to fire, and with the pneumatic apparatus, the products were always charcoal acid, hydro-carbonate gas, and much water, with generally a little empyreumatic oil, and sometimes a trace of prussic acid, leaving a residue varying in weight as just stated.

From the properties above manifested, I conceive I am entitled to declare the black matter, obtained from the bronchial glands, and from the lungs, to be animal charcoal in the uncombined state, i. e. not existing as a constituent ingredient of organized animal solids or fluids.

I mean by the term animal charcoal what is popularly understood. Of course I do not mean pure charcoal. Such a state of this substance cannot here be reasonably expected, either from a consideration of the state of it, as inspired from the atmosphere, or from its necessary impregnation with animal matter during its long residence in the lungs. I imagine no person would hesitate to consider such a coaly substance as the present to be charcoal, if derived from other sources besides the animal economy; it being, as shown by the preceding experiments, a black, tasteless, infusible powder, indissoluble in muriatic acid, nitric acids, and perhaps all common acids, except the sulphuric, affording as large a proportion of charcoal acid as animal and vegetable charcoal which has been exsiccated at the same temperature, and equally resisting fire in close vessels.

For the purposes of physiology, a few theoretical remarks may, perhaps, be useful. I think the charcoal, in the pulmonary organs, is introduced with the air in breathing. In the air, it is suspended in invisibly small particles, derived from the burning of coal, wood, and other inflammable materials in common life. It is admitted that the oxygen of atmospherical air passes through the pulmonary air vesicles, or cells, into the system of blood-vessels; and it is not improbable that through the same channel various matters contained in the air may be introduced. But it is highly reasonable to suppose that the particles of charcoal should be retained in the minutest ramifications of the air tubes, or even in the air vesicles, under various circumstances, to produce the colored appearances on the surface, and in the substance of the lungs, as above described. It must also be considered, that innumerable absorbent lymphatic vessels take their rise in the bronchial tubes; for the lungs are more richly stored with lymphatic vessels than any other organ, excepting the liver. When I compared the black lines and black net-like figures, many of them pentagonal, on the surface of the lungs, with the plates of the lymphatic vessels by Cruickshank, Mascagni, and Fyffe, I found an exact resemblance; and when I found that these vessels contained charcoal, I judged that it was fair to infer, that the lymphatics of the lungs absorb a variety of very different substances, and especially this coaly matter, which they convey to the bronchial glands, and thus render them of a black or dark-blue color. Hereafter, among other enquiries, the color of the large trunks of the lymphatic vessels, just *before* they enter the bronchial glands, and just as they pass out of them, ought to be observed.

served. Also the effect of the charcoal thus conveyed into the thoracic duct, or directly into the blood by the lymphatics from the black glands, is, I presume, worthy of attention.'

According to this theory, we can account reasonably for the absence of the black color of the bronchial glands, and of the lungs in infants, in children, and even frequently in persons of eighteen or twenty years of age. For the same reason these appearances are seldom observed in any brute animal which has fallen under my observation. On a subject so novel, or at least so much neglected, as that on which I am writing, many facts are wanting to establish, demonstratively, any theory which can be proposed; but I know of none, at this time, which are at variance with that I have ventured to offer.

---

*George-street, Hanover-square,*
*June* 13, 1813.

P.S.—Since my last communication to the Society, I have had an opportunity of making some further observations and experiments on this subject.

The lungs of an infant, who lived two days, were obligingly sent to me by my friend Mr. C. M. Clarke. I found the bronchial glands quite white, and the lungs appeared externally of an uniform reddish color. The pulmonary organs of a girl of fifteen years old, I found rather thickly mottled, but the bronchial glands were only tinged on their surface, and did not yield one-fourth of a grain of coaly powder. In two other females also, aged nearly fifteen years, who died of pulmonary consumption, the lungs were not at all marbled, but some of the bronchial glands were tinged black, and others were white. The lungs of two men, the victims of pulmonary phthisis, at the age of twenty-one years, exhibited thinly, black spots, streaks, and areolæ, with many of the glands in question of a deep-blue color. A woman's lungs, thirty-one years of age, were found studded beautifully with black spots and lines, the bronchial glands being all either blackish or blue. In no instance have I observed the lungs and glands, here spoken of, so black, and from which I separated so much charcoal, as in those of a person forty-two years old, whose death was occasioned by most extensively-diffused tubercles, many vomicæ, and a considerable condensation of the pulmonary organs. I now recollect that this subject had been a smoker of tobacco, generally several times, but always once a day, for perhaps more than twenty years. Future observations must determine more satisfactorily the state of the pulmonary organs, according to the impregnation impregnation of the air with sooty vapors. If, hereafter, it be shown that the lungs of persons living remote from sources of such vapors, are still greatly impregnated with coaly matter, the just conclusion can only be, that such matter is more extensively diffused through the atmosphere than is apprehended. This being the fact, it would also afford a proof that it is only the invisibly small particles which are absorbed, for the larger particles remain unabsorbed, entangled in the mucus lining the air vessels, and never get farther, but are rejected from time to time by expectoration. Accordingly, in a morning, healthy people, after the night's rest, very commonly hawk up mucous matter of a bluish color with black streaks, owing to charcoal; and persons in a diseased state, especially by great exertions in coughing, frequently expectorate matter spotted and streaked with black particles. The quantity of coaly matter in the pulmonary organs is not entirely according to the age, for I was disappointed on finding the lungs and glands in a woman of seventy-five years of age, in London, not more deeply colored than is usual at the age of fifty. At present I am unable to state any connection between certain diseases, and the presence of coaly matter.

Farther investigation has shown, that this coaly matter does exist in domesticated brute animals; but as they die, or are killed generally before they attain to twenty years, or even fifteen years of age, the organs in question are seldom seen blackened. However, in diseased conditions, the cases are not according to this rule. With the approbation of Professor Colman and Mr. Sewel, several of the worthy students of the Veterinary College have frequently obliged me by furnishing, for my examination, the lungs of horses and asses. In general, the bronchial glands were merely white, or reddish; but now and then they were partially black. In one instance of an ass, only six months old, these glands were black from coaly matter; but the lungs were uniformly red. The animal had died of peripneumony. In no instance have I seen, in any brute creature, the lungs marbled and streaked with black lines as in the human. There is seldom an opportunity of inspecting horses which die from their natural age, viz. of thirty to forty years; for I am informed they mostly die or are killed in London before they are fifteen or sixteen years old. I have not seen coaly matter in the lungs or glands of the ox kind, sheep, and hogs. The black appearances produced by distended blood-vessels and by ecchymosis, should be recollected, to avoid the error of ascribing them to charcoal. The absence of this matter in human creatures, at the ages just mentioned, when animals are are slaughtered or die, affords a proof, although not a decisive one, that the exemption is more reasonably ascribable to the circumstance of time, and living in the open air, than to the peculiarity of the economy of each species of live being. Consistently with this observation, in the instance of a cat, known to have lived in Mr. Thomas's family at least eighteen years, the bronchial glands were quite black from coaly matter, and the lungs were uniformly red; but in all other examinations of much younger cats, I found these glands either white or red.

The blackness of the lungs from charcoal remains, although hæmorrhage to occasion death has occurred. It is not removable by ablution, or maceration in water, nor by acids, nor alkalies, nor by the early stages of putrefaction. I have not met with a similar coaly substance in any parts of the animal economy except the lungs. The glands of the mesocolon are sometimes black, similarly to the bronchial; but the color soon disappears on immersion in nitric or muriatic acids, no charcoal being separable. The black, or more truly the dark brown tinging liquid of the sepia, I have ascertained by experiments, does not contain uncombined charcoal; this matter existing there only as a constituent ingredient of animal matter.

As I have represented, it is conceived that the coaly matter is very slowly absorbed by the mouths of the lymphatic vessels in the innumerable air tubes and cells.

To determine whether or not this matter exists in these lymphatic vessels, and is the occasion of the black maculæ, streaks, and areolæ, or marbled appearance of the surface of the lungs, I entreated Mr. Wharrie, of St. George's Hospital, whom I knew to be a skilful anatomist, to inject these vessels with quicksilver. In some trials, the injection passed without interruption, in the usual manner; but in others it was apparently obstructed, by meeting with the black lines on the surface. Mr. George Ewbank also, at my desire, very dexterously dissected out about one inch in length of one of these black lines, supposed to be a lymphatic vessel. Being put into a glass capsule, full of nitric acid, the black line immediately was contracted in all dimensions; but it retained its form after digestion, for several days, at a high temperature; afterwards, on gently shaking the capsule, the black line was broken into a number of indissoluble particles. In the interior of the lungs, it is not unusual to see black spots in the middle of tubercles, although these substances consist apparently of self-coagulated lymph probably secreted in the cellular substance, and therefore very likely to envelope lope the coaly matter in the air tubes by the coalescence of numerous minute tubercles.

It has been objected, that the nitric acid may develope the constituent or combined ingredient charcoal of all animal substances, and consequently no proof will be thereby afforded of this matter being extraneous; but on many trials, I have never by this means obtained charcoal from any animal mucilage.

I have no reason to believe, that any of the coaly matter under investigation is dissolved by this acid, for on distilling a pint measure of it from ten grains of the black powder of the bronchial glands, there was no sensible diminution of it, whether it was so treated before ignition, or subsequently: on evaporation to dryness, nitric acid, in which the coaly matter had been boiled, afforded no black sediment. Hence, I conceive that this menstruum may be employed to determine, more accurately and speedily, the proportion of the coaly matter, than any other agent. Sulphuric acid does dissolve a certain portion of this charcoal, affording a transparent liquid, even on dilution with water.—*Phil. Trans. Part II. for* 1813.

